# Metadata mapping and reuse in caBIG™

**DOI:** 10.1186/1471-2105-10-S2-S4

**Published:** 2009-02-05

**Authors:** Isaac Kunz, Ming-Chin Lin, Lewis Frey

**Affiliations:** 1 Department of Biomedical Informatics, School of Medicine, University of Utah, Salt Lake City, UT, USA; 2 Huntsman Cancer Institute, University of Utah, Salt Lake City, UT, USA

## Abstract

**Background:**

This paper proposes that interoperability across biomedical databases can be improved by utilizing a repository of Common Data Elements (CDEs), UML model class-attributes and simple lexical algorithms to facilitate the building domain models. This is examined in the context of an existing system, the National Cancer Institute (NCI)'s cancer Biomedical Informatics Grid (caBIG™). The goal is to demonstrate the deployment of open source tools that can be used to effectively map models and enable the reuse of existing information objects and CDEs in the development of new models for translational research applications. This effort is intended to help developers reuse appropriate CDEs to enable interoperability of their systems when developing within the caBIG™ framework or other frameworks that use metadata repositories.

**Results:**

The Dice (di-grams) and Dynamic algorithms are compared and both algorithms have similar performance matching UML model class-attributes to CDE class object-property pairs. With algorithms used, the baselines for automatically finding the matches are reasonable for the data models examined. It suggests that automatic mapping of UML models and CDEs is feasible within the caBIG™ framework and potentially any framework that uses a metadata repository.

**Conclusion:**

This work opens up the possibility of using mapping algorithms to reduce cost and time required to map local data models to a reference data model such as those used within caBIG™. This effort contributes to facilitating the development of interoperable systems within caBIG™ as well as other metadata frameworks. Such efforts are critical to address the need to develop systems to handle enormous amounts of diverse data that can be leveraged from new biomedical methodologies.

## Introduction

There is a data tsunami of genomic, imaging, proteomic and other high-throughput technologies that is converging upon the field of biomedical informatics. The task at hand is to integrate and synthesise this information into knowledge that can enhance our understanding of biological and clinical systems. The difficulties of channelling such large amounts of data into useful systems often result in sophisticated data structures and data models that strand users on isolated islands of information. Since these data models are created for specific needs at differing institutions, these data models can result in the generation of many heterogeneous data sources and are referred to as data silos [1-3]. These silos are collected, stored, managed, and analyzed using different conceptual representations of the same or similar underlying scientific domains. In essence the data tsunami engenders a multiplicity of tongues due to the creation of isolated islands of information.

Given the many data elements that bench and clinical researchers need to draw on: genomics data, clinical data, expression array data, SNP array data, proteomics data, and more yet to be created; the need for interoperable data sets and systems is becoming paramount. Organizations, researchers, clinicians and ultimately patients will benefit by better integration across data sets and systems [1]. The goal is to enable the interoperability of data and systems by joining data and analyses between organizations to increase the size of the data analyzed and the ease with which research can be replicated. The hope of such interoperable systems is that the speed and impact of the research will be increased.

The purpose of the research presented in this paper is to explore enhancing a metadata infrastructure, such as the National Cancer Institute (NCI)'s cancer Biomedical Informatics Grid (caBIG™), with algorithms that facilitate the creation of interoperable systems. NCI's caBIG™ framework was selected because it utilizes a metadata repository and has terminology curators that work with developers to map models to Common Data Elements (CDEs) and to maintain the interoperability of the caBIG™ framework. Consequently, the algorithms' performance with mapping the models can be compared against the model mappings developed by experts.

## Background

Many industry and research projects require some form of model mapping. Data from one clinic or research facility will not be readable by another unless they have the same data model or a method to translate between the two. The current process to allow such exchanges is costly and time consuming since it requires resources such as database specialists or knowledge engineers to communicate and manually map data elements from one facility to another or to a reference model. Currently this is done manually in a labor-intensive and error-prone process without tools to automate the process [4-6].

This problem promises to worsen in the future as biomedical data rapidly increase due to scientific advancements; particularly with the innovations made in genetic research and molecular biology. For example, UniProt, a universal protein resource that is referenced for many biomedical research projects, reports having to add many new terms and database cross-references [7]. This can result in frequent changes to its model. Another example of changing vocabulary is the NCI vocabulary services that are released monthly to keep information up to date [8]. Manual identification of equivalent model elements consumes time and resources, and may often be the rate-limiting technological step in integrating disparate data sources [9].

Mapping of models is also common in the area of controlled medical vocabularies. Several controlled medical vocabularies (CMVs) are currently available. However, they usually cover diverse domains with different scopes and objectives. The absence of an accepted "standard" method for representing medical concepts, and the need to translate clinical data to existent CMVs has made automated vocabulary mapping an active area of medical informatics research [10]. An accepted method is to map vocabularies to a reference terminology. This eliminates the combinatorial explosion of mappings that would be required otherwise [11]. While the use of a reference terminology is helpful in reducing the cost of mappings by reducing the number of mappings, it is still expensive to map a local model to a reference model. This requires the selection of appropriate metadata components called Common Data Elements (CDEs) that are equivalent between resources that are destined to interoperate.

### caBIG™

caBIG™  is designed as an open source infrastructure that connects resources to enable the sharing of data and tools for cancer research. The NCI launched caBIG™ in 2004 and it includes the development of standards, policies, common applications, and middleware infrastructure to enable more effective sharing of data and research tools. While caBIG™ is designed to provide the framework around use-cases in cancer research, this effort can benefit the entire biomedical informatics community where large-scale data integration becomes a necessity.

The systems developed in the caBIG™ initiative are constructed using a model driven architecture (MDA; ). The MDA approach is used for the construction of well-specified application program interfaces (API) that the grid middleware [4,5] uses to pass semantically and syntactically meaningful data. All data transmitted by the grid is transformed to objects that are derived from models expressed in the Unified Modelling Language (UML) [12,13].

**Figure 1:**
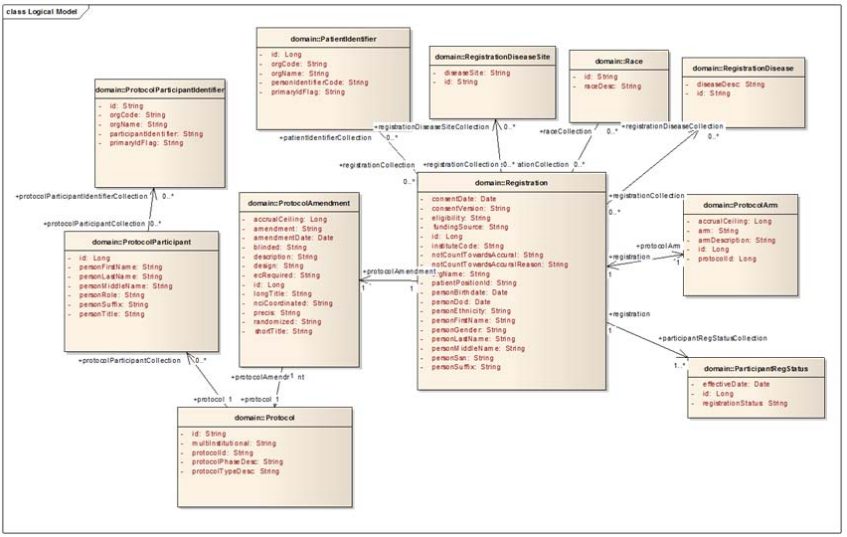
**caBIG™ UML model**. This is an example of a portion of a UML model of a system available in caBIG™. The model describes the classes and attributes of the system and information about function and relationships. Note the class Race has attributes id (a string identifier of a race) and raceDesc (a string description of a race). This Race class is mapped to a CDE within caBIG™ to give a semantic definition and allow reuse of this type of data element.

UML allows developers of resources such as data services and analytical services to describe their services in an abstract manner while constructing meaningful APIs that the grid middleware uses to pass data. UML modelling is used to specify the classes and attributes of the system (See Figure 1).

For systems to interoperate, it is necessary for these two components of the model (i.e., classes and attributes) to be harmonized with identical components in other models across the systems. This paper is examining using lexical matching algorithms to identify the classes and attributes that are common between domain models by mapping to a reference CDE repository.

### Harmonization scaling problem

**Figure 2:**
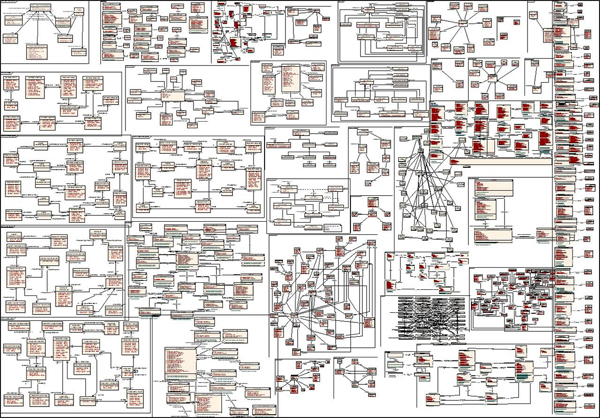
**Visual representation of several caBIG™ UML models**. An example of several UML models available in caBIG™ for reuse.

**Figure 3:**
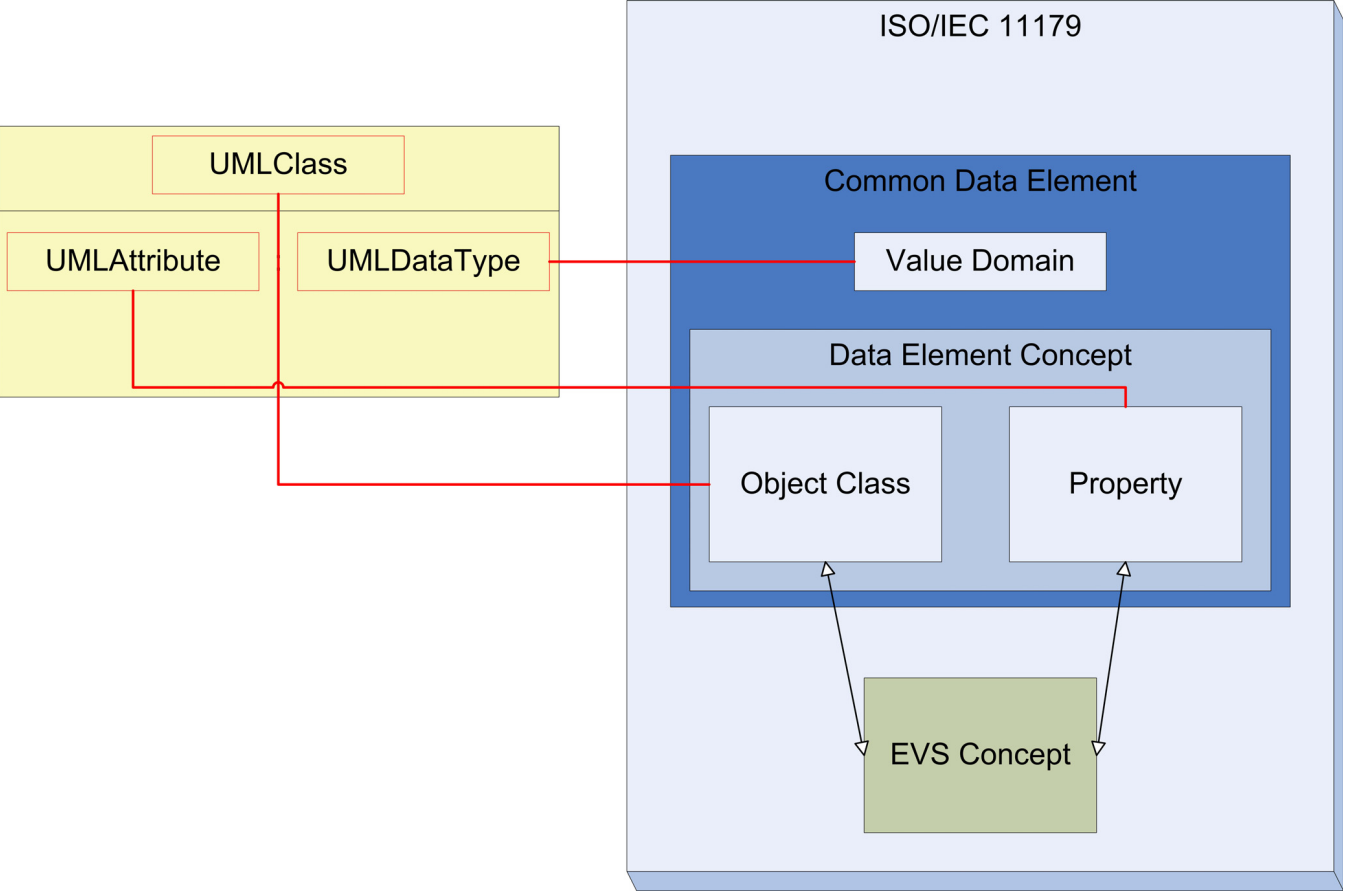
**UML and ISO/IEC11179**. The mapping of UML elements to the ISO 11179 Common Data Elements (CDE) within the caDSR. UML Class maps to Object Class, UML Attribute to Property, and UML Data Type to Property. Object Class and Property components of the Data Element Concept are then mapped to Terminology concepts stored in EVS.

Currently developers go through a manual process of harmonizing new service elements (e.g., UML class-attributes) with those stored in the NCI's Cancer Data Standards Resource (caDSR; metadata repository) in order to achieve interoperability certification of a resource. The caDSR is NCI's implementation of the metadata standard ISO11179 which consists of metadata binding object classes and properties within a data element to controlled terminologies in NCI's Enterprise Vocabulary Service (EVS). Since the space of models within caBIG™ is complex and getting more complex (See Figure 2 for a small subset of the space), the need for tooling to navigate the model space is urgent. In caBIG™, UML models are bound to the components of a CDE with UML classes being bound to object classes, UML attributes being bound to properties and UML data types being bound to value domains (See Figure 3). Consequently, the process of mapping UML models to CDEs in the caDSR is arduous and currently requires an NCI curator (a trained terminologist who is familiar with the NCI model) to work one-on-one with the developer of the new data model to develop the mapping between the UML model class-attributes and the CDEs.

A number of UML models have already gone through this manual mapping processes to CDEs. The difficulties of CDE mapping become even greater with the increasing amount of CDEs available within caDSR and the size of this space is getting larger with every caBIG™ data service or application that is developed.

### Proposed solution to improve scalability

The goal is to mitigate the work involved in reusing CDEs through the reduction of the information an expert is required to examine in order to achieve interoperability and harmonization. In particular, this paper discusses a baseline comparison of two algorithms (di-grams and dynamic programming methodologies) used to map biomedical data models into caBIG™'s CDE space. The question is how close simple lexical algorithms can get to the selection of the appropriate mappings.

The ability of the two algorithms to select the appropriate mapping is also compare across two conditions: Per Project and Combined Project. In practice, developers constrain their UML model comparisons to similar models. This restricted model comparison, referred to as the Per Project condition, restricts the matching of UML class/attribute pairs to the CDEs within the same model space. The Combined Project condition is searching the entire model space. These comparisons are used to explore the feasibility of deploying an open source tool that can be used to map models and enable the reuse of existing information objects and CDEs in the development of new models for translational research applications.

## Methods

In order to map the UML model class-attributes to CDEs, the UML models and the CDEs must be converted to a format that the lexical algorithms can process (Formatting Data Phase). After the data are formatted, data are submitted to each algorithm: Dice's coefficient with di-grams [14] and Dynamic programming using Smith-Waterman's algorithm [15] The algorithms produce similarity ratings that are used to find the best match between the UML model class-attributes to CDE class object-property pairs. To evaluate the goodness of the match, the algorithms' matches are compared to a "Gold Standard" – the matches established through NCI caBIG™ curators. We compare application mappings already in use and currently stored as metadata in the caBIG™ infrastructure by extracting all application UML models and their corresponding CDEs.

### UML model class-attribute data

We tested the algorithms ability to map UML model class-attributes data available from 66 UML models (referred to as projects; see Table 1) to CDE class object-property pairs. In addition, only caBIG™ CDEs that are considered "Released CDE" are used in the mapping. "Released CDEs" are CDEs that have gone through a series of reviews within caBIG™.

**Table 1:** caBIG™ projects/application sizes – 66 UML projects. caBIG™ enabled projects/models used in this research with their corresponding UML element size (class-attribute pairs)

*Project name*	*Size*
Bioconductor 1	75
BiospecimenCoreResource 1	286
BRIDG 1	343
C3PR 1.1	58
C3PR 2	185
CaAERS 1	308
CaArray 2	440
CaArray_1.1	318
caBIO 4	302
CaElmir 1	174
caFE Server 2	80
caGrid 1	14
caIntegrator 2	271
caIntegrator 2.1	328
Caisis 3.5	67
caNano 1	150
caNanoLab	621
Cancer Models Database 2.0	242
Cancer Models Database 2.1	272
Cancer Molecular Pages 1	152
CAP Cancer Checklists 1	194
caTIES 1.0	38
caTIES 2.0	219
caTISSUE CAE 1.2	284
caTISSUE Core 1	287
caTissue_Core 1.1	327
caTissue_Core_1_2	329
caTissue_Core_caArray 1	329
caTRIP Annotation Engine 1	63
CaTRIP Tumor Registry 1	114
CDC NCPHI Proof of Concept .1	9
CGWB 1	91
ChemBank 1	19
Clinical Trials Lab Model 1	84
Clinical Trials Object Data System (CTODS) .53	434
CoCaNUT 1	244
CTMS Metadata Project 1	51
DemoService 1	4
DSD 1	31
GeneConnect 1	59
GenePattern 1	88
Generic Image 1	39
Genomic Identifiers 1	12
geworkbench 1	80
GoMiner 1	69
Grid-enablement of Protein Information Resource (PIR) 1.1	183
Grid-enablement of Protein Information Resource (PIR) 1.2	200
LabKey CPAS Client API 2.1	364
LexBIG 2.2	206
MicroArray Gene Expression Object Model (Mage-OM) 1	140
NCI-60 Drug 1	124
NCI-60 SKY 1	109
NCIA_Model 3	110
NHLBI 1	772
Organism Identification 1	10
PathwayInteractionDatabase 1	59
Patient Study Calendar 2	67
Potential CDEs for Reuse 1	185
ProteomicsLIMS 1	200
Reactome Database Sharing 1	83
RProteomics 1	40
Seed 1	17
SNP500Cancer 1	29
TobaccoInformaticsGrid 1	15
Training Models 1	37
Transcription Annotation Prioritization and Screening System 1	92

### Per project

Each of the 66 UML projects was mapped to a restricted collection of CDEs to which it uses (i.e., restricted to its own model space). This restriction of the search space to corresponding CDEs is reasonable since typically a developer will compare their UML models/projects with similar projects within caBIG™. This condition can be viewed as a curator guided algorithm to mapping models. It is possible to reduce the curator guidance by building an ontology for the models/projects.

### Combined project

Each of the 66 UML project was mapped to the combined set of all the CDEs in the 66 UML models. This condition is more computationally difficult (larger search space) and can be viewed as an automated approach to mapping models.

### Matching UML model class-attributes to CDEs

For both algorithms, the process of matching UML model class-attributes to CDE class object-property pairs consists of two phases: formatting the data and mapping via similarity measures.

### Formatting data phase

The formatting data phase extracts the UML class-attribute pair names and tokenizes them into text strings of words. UML classes and attributes are converted from programming notation to space delimited words. For example the UML attribute "raceDescription" would be converted to "race description."

Next the UMLS Lexical tools lvg2007 API is used to normalize the UML class attribute pairs and the Object Class Property pair of the CDE . The normalization process includes removal of genitives, replacement of punctuation with spaces, removal of stop words, lowercasing words, un-inflection of each word, and word order sorting. This formatting data process produces tokenized strings of UML class/attribute pairs that can be matched to their corresponding object class/property pairs (See Figure 3). Note that only names of the classes and attributes along with the names of the object classes and properties are used.

### Mapping phase

The mapping phase is where Dynamic and Dice's algorithms are applied. The algorithms differ by the similarity measures. For each algorithm, the mapping consists of calculating all the similarity measures between the UML model class-attributes and the CDEs. The similarity scores are rank ordered with the highest similarity scores listed first as likely candidates for the mapping. This is listed on the graphs as percentage of correctly matched CDEs within a given ranking.

### Dice algorithm

Dice's similarity coefficient is a similarity score to measure the lexical similarity [14]. This algorithm requires no knowledge about word formation or semantics and provides resilience to noise (such as abbreviations and misspellings) [10,15]. The algorithm breaks the strings into two letter pairs called di-grams (or N-grams where n equals 2) and then uses Dice's similarity coefficient as follows:

D_fc _= (M × 2) ÷ (S + T) where:

M = number of common elements

S = number of elements from source

T = number of elements from target

### Dynamic algorithm

The Dynamic algorithm is inspired by DNA-sequencing algorithms such as Smith-Waterman [15], a popular edit-distance algorithm. The power of the algorithm comes from its ability to account for gaps in strings where sequences of non-matching characters can be found. The process of comparing the similarity between two strings proceeds by creating a two dimensional matrix where the axes are the strings being compared. Scores are calculated by scanning through each row in the matrix and comparing the letter for the row against the letters in the string at the top of the columns of the matrix. The weighting method gives unique matching score (+8), mismatch score (-8), and gap penalty (-8). The point of the scoring process is to find consecutive sequence of similar substring within the strings being compared. This process is continued until all the scores are calculated in the matrix. Then the algorithms backtrack through the matrix to find a path with the highest score. This score is used to rank the similarity of the two strings.

### "Gold standard"

The "Gold Standard" mappings have been constructed by NCI curators who have created and validated mappings between UML models and CDEs. These existing mappings, serving as our "Gold Standard," are stored in the caDSR and are publicly available for download through the UML Model Browser or by programmatic access via the caDSR API. The caDSR API allows runtime access to metadata, the UML models, and their corresponding mappings to CDEs. This API can be found as part of the caCORE SDK and is publicly available [16].

## Results

**Figure 4:**
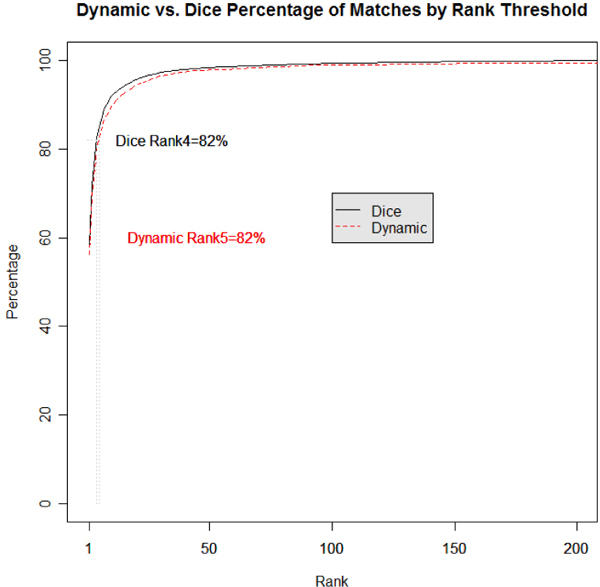
**Per project dice vs dynamic**. Total percentage of "Gold Standard" matches per cumulative rank per project

**Figure 5:**
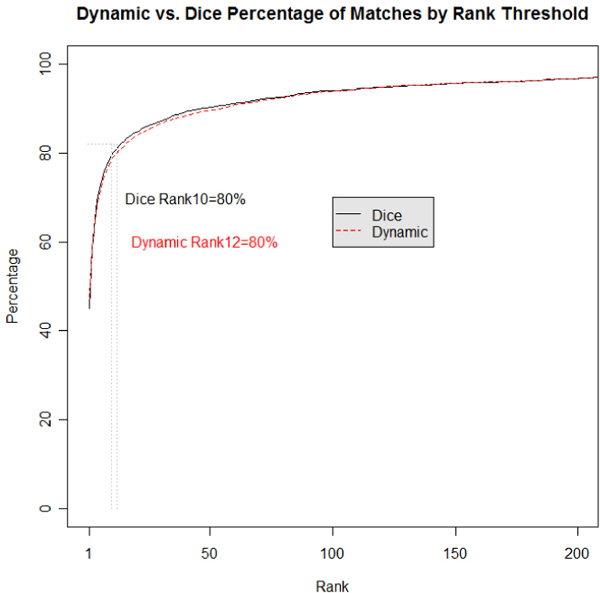
**Combined project dice vs dynamic**. Total percentage of "Gold Standard" matches per cumulative rank for all "RELEASED" CDEs.

The comparisons of the Dice and Dynamic algorithms to the "Gold Standard" are made by plotting the percentage of correct "Gold Standard" matches for each CDE provided by the algorithms. The graphs depicted in Figures 4, 5 are accumulative functions in which the first point corresponds to the percentage of correct matches (e.g., 60%) for a single CDE and the second point corresponds to the total percentage of matches for both the first and second CDE and so forth.

### Per project

Table 2 highlights the percentage of correct "Gold Standard" matches for the Per Project ranking of the Dice and Dynamic algorithms. The Per Project condition only ranks the UML class/attribute pairs against the CDE class object/property pairs within the project. The percent of correct "Gold Standard" matches that occurred in the top rank were 58.2% and 56.3% for the Dice and Dynamic algorithms, respectively. Within the top five ranked matches, 85.1% and 82.6% correctly matched the "Gold Standard" for the Dice and Dynamic algorithms, respectively. The percentages are cumulative and will eventually reach a 100% when all the correct "Gold Standard" matches are within the ranking set. Figure 4 provides the graph of the average percentages for the Per Project condition for all the ranks of the Dice and Dynamic algorithms. As displayed in the figure the results of both algorithms are comparable.

**Table 2:** Per project percentages. Percentage of "Gold Standard" mappings correct in cumulative rankings. For example Dice had 85.1% of the "Gold Standard" mappings returned in the top 5 results.

**Algorithm**	**Rank 1**	**Rank 5**	**Rank 10**	**Rank 25**	**Rank 50**
Dice	58.4	85.1	91.8	96.6	98.3
Dynamic	56.3	82.6	89.5	95.4	97.8

### Combined project

Table 3 highlights the percentage of correct "Gold Standard" matches for the Combined Project condition of the Dice and Dynamic algorithms. The Combined Project condition ranks the UML model class-attribute pairs and CDE class object-property pairs for all projects listed in Table 1. The top rank of the Dice algorithm reaches 45.1% and the Dynamic algorithm reaches 47.6% of the "Gold Standard" correct matches. In the top five ranked matches, 72.1% for the Dice algorithm are correct "Gold Standard" matches while 70.9% for the Dynamic algorithm are correct "Gold Standard" matches. Both algorithms increase to 100% at a slower rate than the Per Project condition. Figure 5 provides the graph of the average percent correct across the Combined Project comparison for all the ranks of the Dice and Dynamic algorithms. As seen from the figure the results of both algorithms are comparable. The cumulative average of Figure 5 does not rise as quickly as the per project cumulative average in Figure 4.

**Table 3:** Combined project percentages. Percentage of "Gold Standard" mappings correct in cumulative rankings. For example Dice had 72.1% of the "Gold Standard" mappings returned in the top 5 results.

**Algorithm**	**Rank 1**	**Rank 5**	**Rank 10**	**Rank 25**	**Rank 50**
Dice	45.1	72.1	79.5	86.1	90.2
Dynamic	47.6	70.9	78.6	85.3	89.6

**Figure 6:**
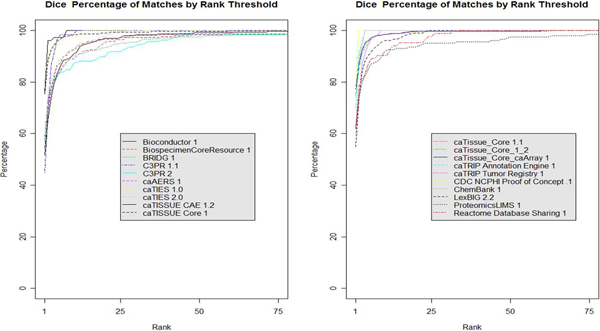
**Dice per project**. This graph shows 20 of the 66 projects mapped to a restricted set of CDEs using the Dice algorithm. Restriction is made by only mapping to corresponding CDEs as indicated in caDSR.

Figure 6 offers a detailed look at the 20 of the 66 projects in how they perform individually for the Dice algorithm in the Per Project condition.

## Discussion

Notice the graphs start with a high percentage of "Gold Standard" matches within the first 5 returned results. This suggests that developers can use the results to help find an appropriate CDE using these automated methods. The class-attribute pairs of the UML models that were analyzed are highly similar to the EVS class-property pairs demonstrating that this could be a valid and effective approach and that mapping of different but similar model types (UML vs. CDE) is feasible.

Figures 4 and 5 illustrate this in terms of the 80-20 rule, where 80% of the gold standard CDE matches are in the top 4 or 5 ranked matches for the Dice and Dynamic algorithm respectively. This would be equivalent of a Google search returning the correct link(s) 80% of the time in the top 4 or 5 listed links. Since currently searching for CDEs to reuse is very labor intensive this can reduce roughly 80% of that work simply by matching developer models against the correct project. Since developers are aware of the domain they are developing systems within, it is reasonable to expect them to compare proteomic models against other proteomic models in the repository (i.e., Per Project comparison) instead of comparing them against tissue banking models or the entire set of models (i.e., Combine Project comparison).

Comparing against the combined models space, the performance of the algorithms degrade somewhat. Given the simple nature of the lexical matching algorithms, they perform relatively well in the Combined Project condition. Still the results suggest that a tool to help the developers navigate the model space would facilitate identifying a higher number of correct matches. The findings from the Combined Project comparison point to the need for an ontological space of models. This will help the developer navigate the space in order to identify the correct model to compare his or her UML class/attributes against or one that algorithms could utilize to constrain the comparison space.

Both Dice and Dynamic algorithms have their own strengths. Dice is relatively simple and not as computationally intensive as dynamic programming. Dynamic programming requires tuning of the scoring variables such as gap scores and adjusting the gap penalty for large gaps in the strings where mismatches are found. It is capable of using longer sequences compared to di-grams; although for this task this feature does not appear to be necessary.

### Caveats

A look at the datasets in Figure 6 shows that some UML model mappings performed better than others. As an example we look at caTissue CORE, caArray and Proteomics LIMS, with 329 and 200 UML class-attribute pairs respectively because they are similar in size with acceptable but differing mapping performances. caTissue has 96 percent of the correct matches returned within the top 5 ranks while ProtLIMS has 85 percent (see Table 4). By looking at the mappings that performed poorly we can improve our algorithms as well as create guidelines for improving automated mapping.

**Table 4:** Dice caTissue CORE caArray and proteomics LIMS. caTissue CORE caArray and Proteomics LIMS percentage of "Gold Standard" mappings correct in cumulative rankings. Differences in mapping scores illustrate various levels of UML class-attribute alignments with CDE class-properties.

**Project Name**	**Rank 1**	**Rank 5**	**Rank 10**	**Rank 25**	**Rank 50**
CaTissue_Core_caArray	77.2	**96.0**	98.5	99.7	99.7
ProteomicsLIMS	62	**85**	91.5	95	97.5

The Dice algorithm did have difficulty with some of the matches. Table 5 shows a comparison of the UML class-attribute and its corresponding "Gold Standard" mapping for caTissue CORE caArray and Proteomics LIMS. These two were chosen to compare because their similarity in size. Mapping performance of our implementation of the Dice algorithm appears to be reduced when abbreviations and synonyms are used. For example Protlims mapping of the UML class-attribute "Sample.label" is pre-processed and converted to "label sample" while the CDE class-property "Specimen Tracer" (short name 2519354v1.0:2178533v1.0) is formatted and converted to "specimen tracer". The current implementation of the Dice algorithm doesn't score this true mapping well when it should have the highest mapping score. The reason for this poor performance appears to be due to the inability to resolve synonyms and determine that specimen and sample are actually the same and that a tracer refers to a label. Another challenge illustrated in Table 5 is difficulty in resolving abbreviations involving numbers. The Dice algorithm is unable to resolve the similarity between "gel2d id sample" to "2 dimensional electrophoresis gel identifier". The gel2d word is not broken down into three separate words as it should. Also from Table 5 we see in places where words are duplicated exacerbating the effects of the algorithms inability to resolve synonyms. Protlims mapping of the UML class-attribute SampleType.sampleTypeId is pre-processed and converted to "id sample sample type type" while the CDE class-property Type Specimen Identifier (short name 2422846v1.0:2178534v1.0) is pre-processed and converted to "identifier specimen type".

**Table 5:** Difficult matches. caTissue and ProtLIMS UML class-attribute compared to CDE class-property pairs are shown here where the dice algorithm scored lower than expected. Reduced performance of the algorithms tends to occur when abbreviations and synonyms appear. For example ProtLIMS gel2d is used in UML to represent 2 dimensional electrophoresis gel.

**caTISSUE CORE caArray (size 329)**		**ProteomicsLIMS (size 200)**	
	
**UML**	**CDE**	**UML**	**CDE**
	
distribute id item	distribution identifier specimen	label sample	specimen tracer
biohazard id	biohazardous identifier substance	identification sample	name specimen
csm id user user	common identifier module security user user	gel2d id sample	2 dimensional electrophoresis gel identifier
id site	identifier site	id plate plate sample sample	identifier microplate
check check event id out parameter	identifier object parameter present remove status	gel2d identification	2 dimensional electrophoresis gel name
numb participant security social	participant ssn	id log log sample sample	identifier log quantity specimen
container id storage	identifier storage unit	file file id lim lim	file identifier information laboratory management system
audit event id user	audit event login name	id sample sample type type	identifier specimen type
date start user	begin date user	id raw sample sample	identifier raw specimen

With adjustments it is likely we will improve both the algorithms' performance. Adjustments could be made to the parameters of each algorithm as well as modifying normalization techniques. Normalization techniques can hurt or help each algorithm depending upon the properties of each model such as duplicate words and which stop words to remove. We chose to go with the default normalization method used in the UMLS API. While both algorithms have similar performance dynamic programming is considerably more computationally intensive, requiring more memory and time to execute, and therefore we would recommend using the faster method of Dice over Dynamic when comparing only names.

The results show that names of UML class-attributes match well with CDE class-properties. It is possible that this is an artifact of the mapping process between the UML models and the EVS concepts. Due to the process and difficulty of the manual mapping the developers may have named their UML elements similar to the EVS concepts.

We have shown the possibility of approaching this problem of mapping UML using lexical algorithms. Given the simplicity of the approach taken, the number of matches is surprising. The mapping results suggest that the mapping processes could at least be partially automated. Developers could iteratively identify reusable CDEs and correctly identify around 80% with relatively small ranked sets when reducing the search space of CDEs choosing a similar model space to work in. This would be an improvement over the current manual mapping process.

Verification will still be need to be part of the caBIG™ review process to ensure accurate mapping but this type of mapping tool could be used by developers as well as by reviewers to hasten the process. While this leads to a mapping process that is not entirely automated, researchers such as Sheth and Larson have assumed that automated mapping is not accurate enough to be used un-supervised by a human[17]. Thus, a tool that facilitates mapping UML models to CDEs is a realistic approach to mapping models in the biomedical informatics domain.

## Future work

We believe that applying semantic techniques to this problem will further enhance the usefulness of this type of mapping tool as indicated by other mapping efforts [4-6,9,18-20]. Future goals are to include semantic mapping tools of UMLS. UMLS have tools that can analyze text and return UMLS concepts. We plan to map UML model descriptions and names into UMLS concepts and then use the mapping stored in EVS to convert to EVS concepts. These concepts will be used to search the EVS for CDEs that contain them and then returned to the user as candidates. The challenge of mapping two models is commonly addressed by lexical methods, logical methods[4], and a hybrid of both [20,21].

The Dynamic scoring method performs well in our preliminary investigation, but it can potentially be improved by creating a substitution matrix for assigning different mismatch scores according to different substitution or assigning less of a penalty score when having continuous gaps.

The long-term goal of this research is to produce an open source tool that has a broad application for mapping ontologies, data models, and/or terminologies. This tool will implement the current state of the art mapping algorithms. In addition to developing this tool for comparing current mapping algorithms it will serve as at test bed for the development of new algorithms or hybrid algorithms that combine the techniques.

## Conclusion

This effort contributes to the creation of interoperable systems within caBIG™ and other similar frameworks. The Dice and Dynamic algorithms are compared and both algorithms have similar performance. Results of this study demonstrate that the names of the UML elements (class name and attribute name) can be used effectively to map to existing CDEs. The lexical matching algorithms can facilitate the reuse of CDEs and reduce the work that needs to be done by a curator to identify pre-existing CDEs that match developers UML class/attribute pairs. It suggests that automatic mapping of UML models and CDEs are feasible within caBIG™ as well as other metadata frameworks.

## Competing interests

This work was funded in part by the NCI caBIG™ initiative for which LF is the PI at the University of Utah Huntsman Cancer Institute. LF receives funding from the NCI to participate in the following caBIG™ workgroups: Vocabulary and Common Data Elements (VDCE), Population Science (PopSci) and Documentation and Training. LF is also funded to serve as a VCDE guide to mentors. LF has used some of this funding to pay IK as a graduate research assistant.

## Authors' contributions

LF came up with the concept and design of the approach taken for mapping metadata. IK implemented the algorithms and collected data. IK drafted the manuscript, created tables and figures and participated in the revision. ML contributed the implementation and tuning of the dynamic algorithms. LF, ML, IK made many contributions to the analysis of the data and interpretation of the results. All authors read and approved the final manuscript.
